# Technical Note: Nuclear imaging with an x‐ray flat panel detector: A proof‐of‐concept study

**DOI:** 10.1002/mp.14191

**Published:** 2020-05-08

**Authors:** Martijn M. A. Dietze, Wilco J. C. Koppert, Rob van Rooij, Hugo W. A. M. de Jong

**Affiliations:** ^1^ Radiology and Nuclear Medicine Utrecht University and University Medical Center Utrecht P.O. Box 85500 3508 GA Utrecht Netherlands; ^2^ Image Sciences Institute Utrecht University and University Medical Center Utrecht P.O. Box 85500 3508 GA Utrecht Netherlands

**Keywords:** CBCT, collimator, flat panel, Gamma camera, SPECT

## Abstract

**Purpose:**

Interventional procedures involving radionuclides (e.g., radioembolization) would benefit from single‐photon emission computed tomography (SPECT) performed in the intervention room because the activity distribution could be immediately visualized. We believe it might be possible to perform SPECT with the C‐arm cone beam computed tomography (CBCT) scanner present in the intervention room by equipping the x‐ray flat panel detector with a collimator. The purpose of this study is to demonstrate the approach and to investigate the achievable SPECT reconstruction quality.

**Methods:**

A proof‐of‐concept experiment was performed to evaluate the possibility of nuclear imaging with an x‐ray flat panel detector. The experiment was digitally replicated to study the accuracy of the simulations. Three flat panel configurations (with standard hardware and reconstruction methodology, with sophisticated reconstruction methodology, and with expected future hardware) and a conventional gamma camera were evaluated. The Jaszczak and the NEMA IQ phantom (filled with ^99m^Tc) were simulated and assessed on resolution and contrast‐to‐noise ratio (CNR).

**Results:**

The proof‐of‐concept experiment demonstrated that nuclear images could be obtained from the flat panel detector. The simulation of the same configuration demonstrated that simulations could accurately predict the flat panel detector response. The CNR of the 37 mm sphere in the NEMA IQ phantom was 22.8 ± 1.2 for the gamma camera reconstructions, while it was 11.3 ± 0.7 for the standard flat panel detector. With sophisticated reconstruction methodology, the CNR improved to 13.5 ± 1.4. The CNR can be expected to advance to 18.1 ± 1.3 for future flat panel detectors.

**Conclusions:**

The x‐ray flat panel detector of a CBCT scanner might be used to perform nuclear imaging. The SPECT reconstruction quality will be lower than that achieved by a conventional gamma camera. The flat panel detector approach could, however, be useful in providing a cost‐effective alternative to the purchase of a mobile SPECT scanner for enabling interventional scanning.

## 1 INTRODUCTION

Interventional procedures involving radionuclides (e.g., radioembolization) would benefit from single‐photon emission computed tomography (SPECT) performed in the intervention room because this would allow the activity distribution to be immediately visualized. Work is ongoing on the development of mobile and compact SPECT scanners.^1^, ^2^, ^3^ However, the associated costs of a new scanner might raise a threshold for widespread application in clinical practice. It would be beneficial if a method for interventional SPECT scanning is developed which does not require the purchase of an additional scanner.

A scanner that is usually already available in the intervention room is the C‐arm cone beam computed tomography (CBCT) scanner, which is used, for example, for two‐dimensional (2D) fluoroscopic guidance and three‐dimensional (3D) position verification.^4^ Although the CBCT scanner design is optimized for imaging of x‐rays, its flat panel detector should also be able to detect gamma photons when equipped with a collimator. The difference with a conventional gamma camera is that the detecting efficiency of the flat panel detector will be much lower and that currently no photon counting and thus photon energy selection can be performed; its intrinsic spatial resolution is, however, much better.

Due to the lower detector sensitivity, the SPECT reconstructions from a flat panel detector can be expected to be noisier than those from a conventional gamma camera. We propose a combination of two methods to boost the flat panel detector sensitivity. First, since the flat panel detector has an excellent intrinsic spatial resolution, the reconstruction resolution can be improved by incorporating a detailed collimator model. This methodology has been shown to be feasible in other studies utilizing small pixel size detectors.^5^, ^6^, ^7^, ^8^ Second, the improved resolution can be traded with a higher sensitivity by enlarging the collimator hole width. The combination of these methods should be able to improve the reconstruction quality.

This study starts by illustrating the feasibility of nuclear imaging with a flat panel detector using a proof‐of‐concept experiment. Using simulations, the achievable reconstruction quality of a flat panel detector is evaluated and compared with that of a conventional gamma camera. It is studied whether the combination of detailed collimator modeling and enlarged collimator holes provides improvements to the reconstruction quality. Finally, the reconstruction quality of future flat panel detectors (which are expected to possess the capability for photon energy selection^9^, ^10^) is assessed.

## 2 MATERIALS AND METHODS

### 2.A. Proof‐of‐concept experiment

An experiment was performed to illustrate the feasibility of nuclear imaging with an x‐ray flat panel detector. A parallel‐hole collimator (29.1 mm hole length, 2.49 mm hole width, 0.50 mm septal thickness) was positioned directly in front of a commercially available flat panel detector with 0.308 mm isotropic pixel size (Pixium 3040; Trixell, Moirans, France). The flat panel detector used a thin CsI crystal for scintillation and had an energy‐integrating readout. A bottle (12 mm inner radius) filled with 414 MBq ^99m^Tc was positioned 12 cm in front of the flat panel detector. A projection was made with a measurement time of 50 s.

The experiment configuration was then digitally replicated in GATE^11^ (with photoelectric effect, Compton scatter, and Rayleigh scatter in the source and the collimator) to study to what extent the simulations can accurately predict the flat panel detector response. The acquired projections from the real data and the simulation were compared on their shape and size.

### 2.B. Detailed collimator modeling

**Fig. 1:**
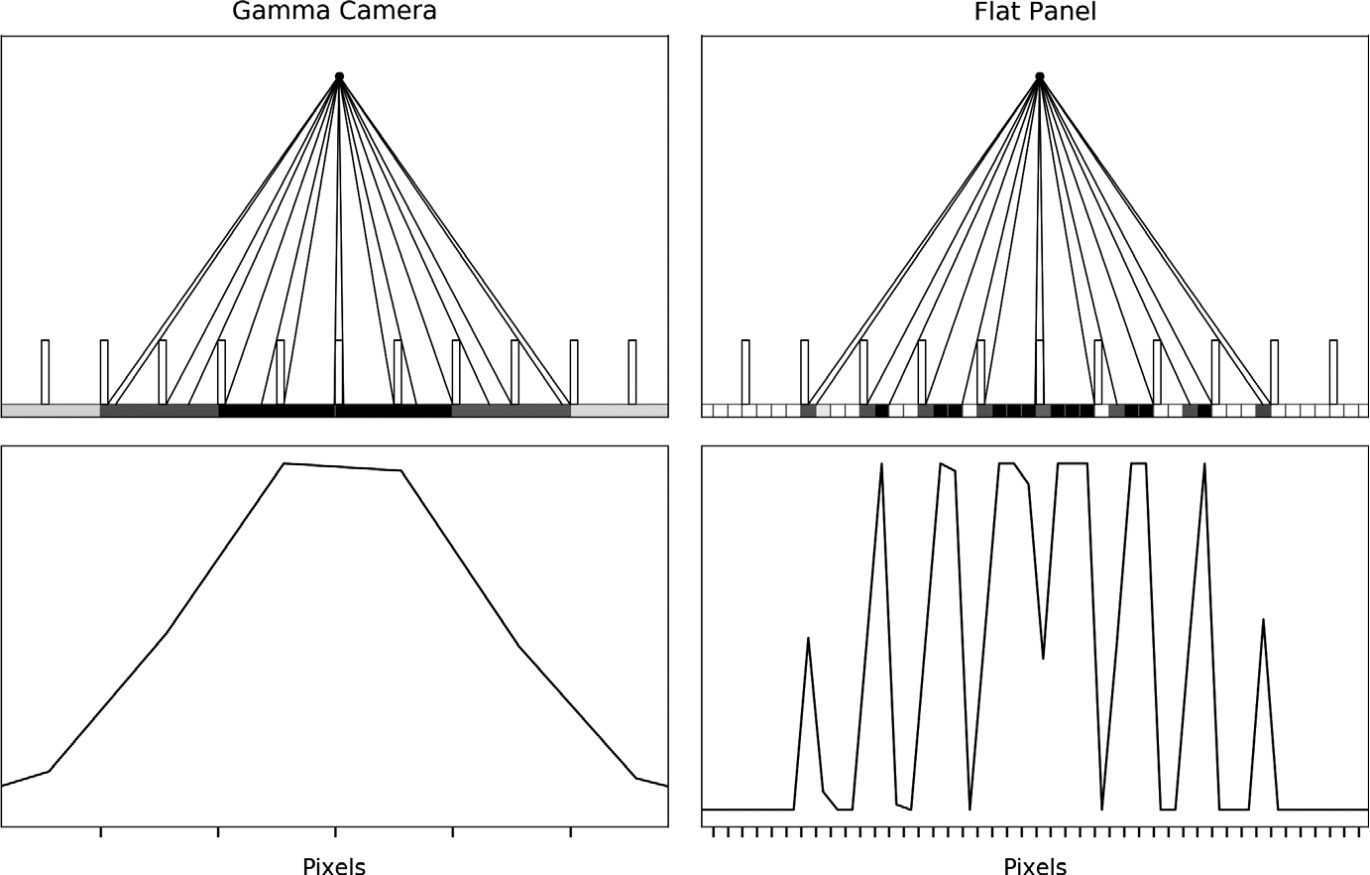
The profiles obtained from a point source positioned 10 cm in front of the collimator for a conventional gamma camera and a flat panel detector. The black lines in the top figures denote the optical transmission of photons through the collimator.

Figure 1 illustrates the projection geometry of a point source positioned 10 cm in front of a low‐energy parallel‐hole collimator (24.05 mm hole length, 1.077 mm hole width, 0.155 mm septal thickness) for a conventional gamma camera (with a pixel size of 2.464 mm) and a flat panel detector (pixel size of 0.308 mm). The profile of the flat panel detector projection is very different from that of the conventional gamma camera because the small pixel size allows to resolve patterns that are smaller than the distance between the septa. Additional resolution information is retrieved from the position and the magnitude of the peaks present in the detector profile.

To take the additional resolution information fully into account in the reconstruction, accurate point spread function (PSF) models are required in both the forward projection of the reconstructor (i.e., given a source in the reconstruction space, this is the resulting profile on the detector) as well as the backward projection (i.e., given a count on the detector, these are the locations in the reconstruction space where it could have originated from). Practically, this means that the PSF models used in the reconstructor should be a function of the relative position above the collimator septum (in addition to being depth dependent). We will refer to this method as “detailed collimator modeling”.

### 2.C. Point spread function generation

**Fig. 2:**
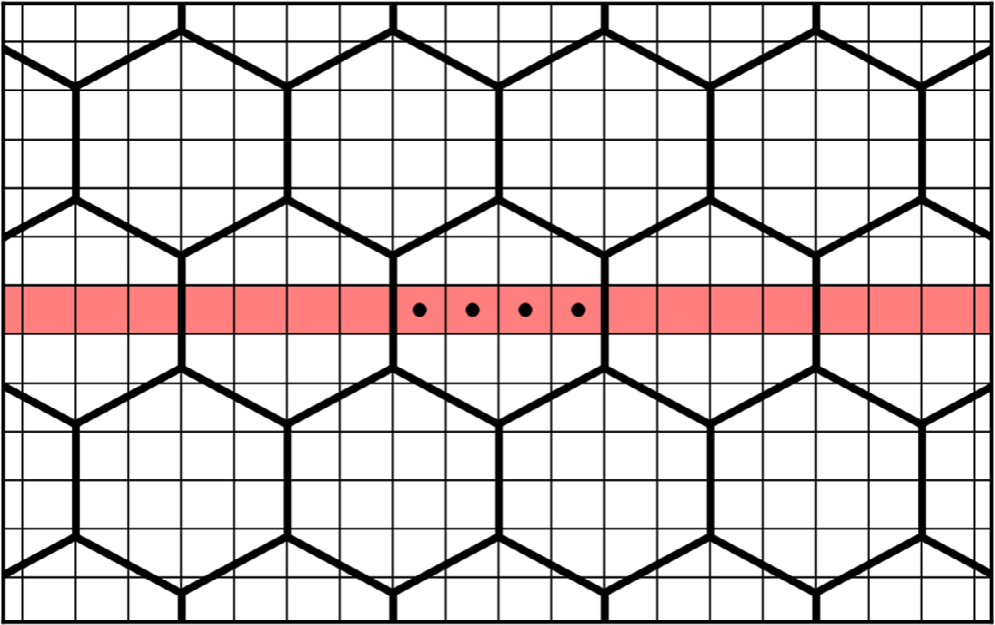
Top view of the custom low‐energy high‐resolution collimator in front of the flat panel detector pixels. The black dots denote the locations where the point spread function models are sampled. Marked in red is the slice that is used in the two‐dimensional reconstruction. [Color figure can be viewed at wileyonlinelibrary.com]

The detailed collimator modeling requires a separate PSF model for every individual pixel in the reconstruction space, which is computationally demanding to simulate. To reduce the number of samples required, we will make use of the repeating pattern of a parallel‐hole collimator. A simulated low‐energy high‐resolution (LEHR) collimator (hole width of 1.270 mm^12^, ^14^) had its dimensions slightly reduced to obtain a hole width of 1.232 mm (this resized collimator will be referred to as the “custom LEHR”). Exactly four detector pixels (with a size of 0.308 mm) now fit into a collimator hole (see Fig. 2). Only those pixels need to be sampled since the remainder of the space is covered by repetition (see the black dots in Fig. 2).

Reconstructions were performed in 2D (rather than 3D) to reduce the computation time even further. Only the slice that is positioned over the middle of the collimator holes (see the pixels marked in red in Fig. 2) was used in the reconstruction and hence only the PSF models for this specific slice were generated. Reconstruction of other slices would require additional PSF models.

The PSF models of the forward projector were created by simulating ^99m^Tc point sources in GATE, positioned above the locations marked in Fig. 2 at distances from the detector ranging from 0 to 40 cm (in steps of 4.6 mm). The simulations were made as realistic as possible by including the parallax effect, signal mixing, collimator attenuation and scatter, and septal penetration. The models were created for the flat panel detector as well as a conventional gamma camera (based on the Siemens Symbia T16 scanner). The intrinsic spatial resolution of the flat panel detector was assumed to be smaller than the detector pixel size. For the conventional gamma camera, a 3.8 mm full width at half maximum (FWHM) intrinsic spatial resolution was applied.^12^, ^14^

The PSF models of the backward projector were created by positioning point sources of ^99m^Tc at the detector pixel locations and then tracing their backward path through the collimator. This results in a matrix with the potential source locations that can be included in the back projection step of the reconstructor.

### 2.D. Collimator design

In the design of a parallel‐hole collimator, there exists a trade‐off between sensitivity and resolution: by enlarging the collimator hole width, the detector sensitivity can be boosted at the expense of its resolution. Hence, in addition to the custom LEHR collimator (of four‐pixel hole width), a collimator with enlarged holes (with eight‐pixel hole width) was also simulated. For every configuration, the septal thickness was set such that the septal penetration was 1% (see Table I for the obtained specific dimensions).^13^, ^15^

**Table I:** Dimensions of the studied collimators.

Collimator	Pixels per hole	Hole length (mm)	Hole width (mm)	Septal thickness (mm)
LEHR	4.12	24.05	1.110	0.160
Custom LEHR	4	24.05	1.077	0.155
Enlarged holes	8	24.05	2.154	0.310

Abbreviation: LEHR, low‐energy high‐resolution.

### 2.E. Reconstruction

A simulation study was performed to study the reconstruction resolution and noise characteristics of the detector configurations. For every configuration, the detector simulated a body‐tracing orbit with a 1 cm phantom‐detector gap. Projections were created with 120 angles over 360° and the reconstruction used the OSEM algorithm with ten iterations and eight subsets. The distributions were reconstructed on a 1496 × 1496 grid with 0.308 mm isotropic pixel size.

Photon attenuation was simulated using the accompanying attenuation maps. Photon scatter was challenging to implement since the reconstruction was performed in 2D. To achieve a realistic scatter estimate, the 2D slice was during the reconstruction temporarily transformed to a down‐sampled symmetric 3D volume (136 × 136 × 70 grid with 3.388 mm isotropic voxel size) from which the scatter contribution to the 2D slice was calculated. The scattered photons were simulated using the Utrecht Monte Carlo System (UMCS) simulator.^12^, ^13^, ^14^, ^15^ This methodology is valid for the phantoms studied in this work since they have an attenuation map that is symmetric in height.

The reconstructions were made with or without detailed collimator modeling depending on the detector configuration. In the case of no detailed collimator modeling, only the depth dependence of the PSF models was taken into account (as is done in the majority of SPECT reconstructors). Similarly, the reconstructions were made with or without the capability for energy selection. In the case of energy selection, only the photopeak window (from 130 to 150 keV) was used. In the case of no energy selection, the entire energy spectrum was summed together.

Four detector configurations were simulated. The reference reconstruction for comparison purposes was the *Gamma camera* (custom LEHR collimator, no detailed collimator modeling, with energy selection). This option illustrates the performance that can currently be achieved in the clinic. For the flat panel detector, the following three configurations were simulated:

− *Flat panel detector — Standard* (custom LEHR collimator, no detailed collimator modeling, no energy selection). This configuration illustrates the reconstruction performance if one uses the conventional reconstruction methodology for the currently used flat panel detector.
− *Flat panel detector — With Modifications* (enlarged collimator holes, with detailed collimator modeling, no energy selection). This configuration employs detailed collimator modeling to utilize the excellent intrinsic spatial resolution of the flat panel detector to obtain improved reconstruction resolution. The improved reconstruction resolution is hence traded with an improved detector sensitivity by enlarging the collimator holes in order to reduce the reconstruction noise.
− *Flat panel detector* — *Future* (enlarged collimator holes, with detailed collimator modeling, with energy selection). This configuration illustrates which reconstruction quality might be achieved for future flat panel detectors with the capability for photon energy selection.

Two distributions were simulated to assess the performance of the detector configurations:

− The Jaszczak phantom (consisting of a circular disk with cold rods of 4.8, 6.4, 7.9, 9.5, 11.1, and 12.7 mm in diameter) without Poisson noise. The average contrast of the 7.9 mm spheres was calculated as a measure of the reconstruction resolution.
− The NEMA IQ phantom (consisting of a background compartment with hot spheres of 10, 13, 17, 22, 28, and 37 mm in diameter added at an 8:1 ratio) with Poisson noise equivalent to 1000 MBq total phantom activity at a 4.79 mm slice reconstruction with 30 s per projection measurement time. A postreconstruction Gaussian filter of 5 mm FWHM was applied to smooth the reconstructions. Ten noise realizations were performed for every detector configuration to study the stability of the reconstructions. The maximum CNR of the 37 mm sphere was calculated (with the background compartment eroded by 5 mm) as a measure of the reconstruction quality.

## 3 RESULTS

### 3.A. Proof‐of‐concept experiment

**Fig. 3:**
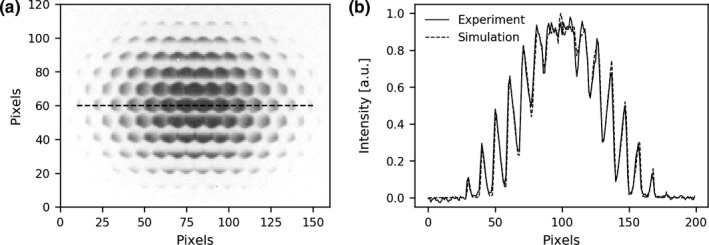
(a) Projection image obtained from the proof‐of‐concept experiment. (b) Line profile corresponding to the dashed line in the projection image, in arbitrary units (a.u.) together with the profile obtained from the simulation.

The projection image obtained from the proof‐of‐concept experiment is illustrated in Fig. 3(a). Since the acquisition time was long and the source activity was relatively high, an almost noise‐less projection was obtained and the collimator structure can be easily observed in the image. The profile on the dashed line is shown together with the profile obtained from the simulation of the experiment in Fig. 3(b). The agreement between the profiles indicates that the simulations are able to accurately model the physical measurements.

### 3.B. Reconstruction

#### 3.B.1. Gamma camera

**Fig. 4:**
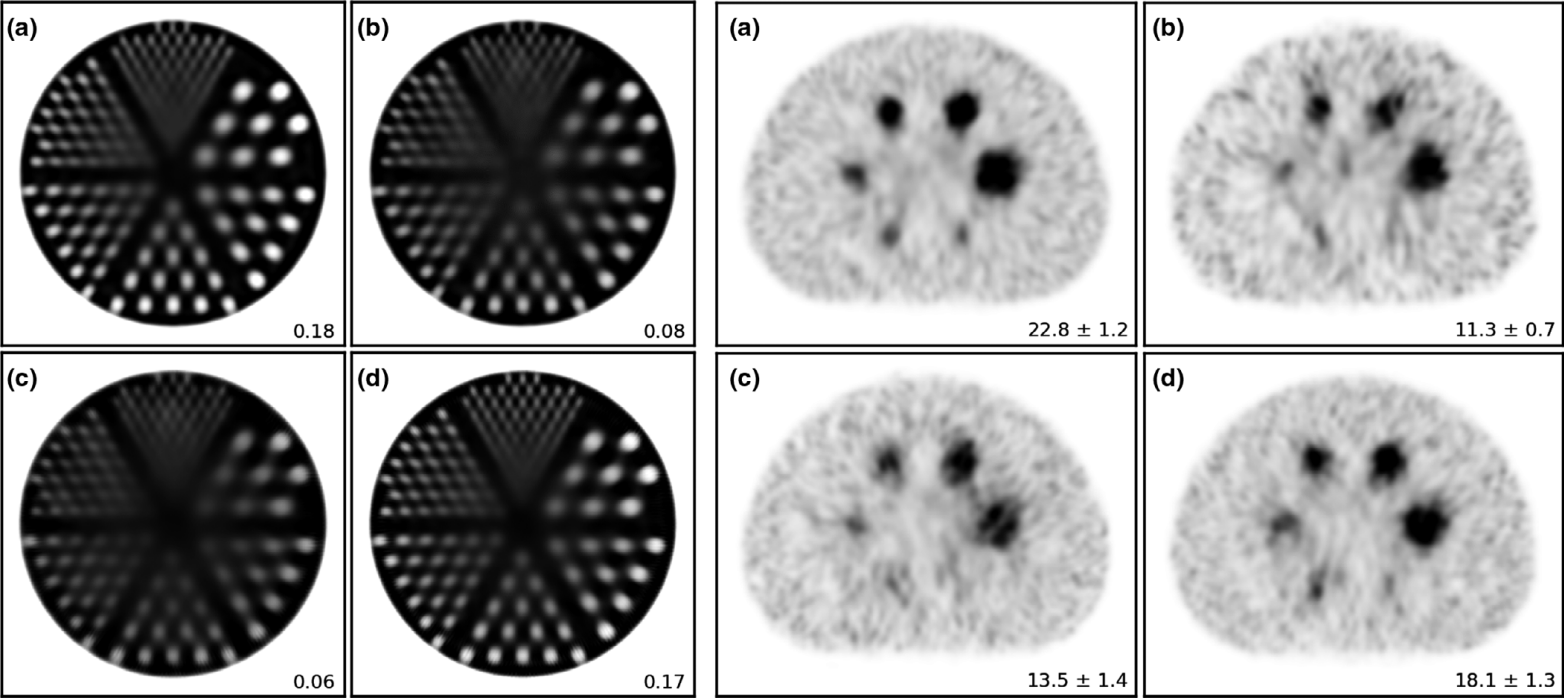
Reconstructions of the Jaszczak phantom and NEMA IQ phantom obtained from the (a) conventional gamma camera, (b) standard flat panel detector, (c) flat panel detector with modifications, and (d) future flat panel detector. For the Jaszczak phantom, the values reported in the bottom right represent the average contrast over the 7.9 mm rods. For the NEMA IQ phantom, they represent the maximum contrast‐to‐noise ratio of the 37 mm sphere averaged over the ten noise realizations.

The reconstructions obtained from the conventional gamma camera (custom LEHR collimator, no detailed collimator modeling, with energy selection) are shown in Fig. 4(a). The performance of this detector configuration can be currently achieved in the clinic and may be used as a reference to compare the quality of the flat panel reconstructions with.

#### 3.B.2. Flat panel detector — Standard

The reconstructions obtained from the currently used flat panel detector when using conventional reconstruction technology (custom LEHR collimator, no detailed collimator modeling, no energy selection) are shown in Fig. 4(b). The contrast in the Jaszczak phantom was lower than that achieved by the gamma camera due to scattered photons (since no photon energy selection was available). The CNR in the NEMA IQ phantom was lower than that of the gamma camera because of the scattered photons and the relatively lower detector sensitivity.

#### 3.B.3. Flat panel detector — With modifications

The reconstructions obtained from the flat panel detector with modifications (enlarged collimator holes, with detailed collimator modeling, no energy selection) are shown in Fig. 4(c). The contrast obtained in the Jaszczak phantom was similar to that achieved with the standard flat panel detector, which indicates that the detailed collimator modeling was able to accurately maintain the reconstruction contrast when moving to a lower‐resolution collimator. The CNR in the NEMA IQ phantom improved when compared with the standard flat panel detector due to the relatively higher detector sensitivity.

#### 3.B.4. Flat panel detector — Future

The reconstructions obtained from the future flat panel detector (enlarged collimator holes, with detailed collimator modeling, with energy selection) are shown in Fig. 4(d). By making use of the photon energy selection capability, most scattered photons could now be accurately discarded. The contrast in the Jaszczak phantom became similar to that obtained from the gamma camera. The CNR in the NEMA IQ phantom improved considerably over that achieved by the flat panel with modifications and approached the value obtained by the conventional gamma camera.

## 4 DISCUSSION

Interventional procedures involving radionuclides would benefit from SPECT performed in the intervention room because the activity distribution could be immediately visualized. This study demonstrated the achievable SPECT reconstruction with the x‐ray flat panel of the C‐arm CBCT scanner when using current technology, when making modifications to the collimator design and the reconstruction methodology, and when using projected future technology.

It is unknown which image quality will be required for interventional SPECT since such guided procedures have not yet been performed. However, some assumptions can be made based on current clinical care. For instance in radioembolization (which is of one the proposed applications for interventional SPECT scanning), it has been demonstrated that various quantitative measures (e.g., lung‐shunting fraction, tumor uptake ratio) can be accurately determined down to 1/10th of the clinically used number of counts.^16^, ^17^, ^18^ These findings suggest that the flat panel detector (which similarly has a lower count rate) should also be able to retrieve accurate clinical results.

Unfortunately, it was not possible to perform SPECT with the proof‐of‐concept experiment because our approach required a pixel‐matched collimator, which was not available. Therefore, this study was mainly intended as an initial exploration of the combined SPECT and CBCT scanner design. Using the results from this work as a reference, the next step would be to produce a pixel‐matched collimator and to illustrate the feasibility of SPECT on a flat panel detector with physical phantom experiments.

The generation of the PSF models and the MLEM reconstructions are computationally very demanding. For these reasons, we chose to use employ a pixel‐matched collimator and to only reconstruct in 2D. There are no physical limitations in using other collimators or reconstructing in 3D except for the substantial increase in computational time. Variance reduction techniques and multiprocessing can improve the speed of both the generation of the PSF models and the MLEM reconstruction. We recommend using square‐shaped pixel‐matched collimators (as applied in CZT cameras) for future studies since this collimator design is symmetric in both directions and hence reduces the number of required PSF models.

The collimator needs to be designed very precisely for the detailed collimator modeling to function. Current lead collimators tend to possess small manufacturing errors, which makes their response unpredictable at times. There is, however, an increasing trend of manufacturing collimators via 3D printing. These printers can use more dense materials (e.g., tungsten) and can very precisely construct the collimator. We thus believe that collimator manufacturing will not be a limiting factor for the presented approach.

Similar to the above argument, the location of the collimator relative to the detector needs to be known with high precision. In the setting of interventional SPECT, the collimator would be placed on the CBCT scanner after the regular interventional procedure is finished. An x‐ray shot could then be made to determine the collimator position relative to the flat panel detector.

The majority of CBCT scanners perform a circular orbit around the patient. For SPECT, however, it is desirable to perform a body‐tracing orbit so that the best resolution is achieved. This would require some mechanical adjustments to the scanner design, but these are not expected to be limiting because most CBCT scanners already have large freedom in their movement. The extra weight introduced by the collimator might, however, require stronger motors for the detector rotation.

Summarizing the above points: although we have shown that it would be technically feasible to perform nuclear imaging with a flat panel detector, it is not expected that current CBCT scanners can be immediately used to perform interventional SPECT with. Most of the mentioned challenges should, however, be resolvable by either more computational power or small modifications to the CBCT scanner design. We thus believe it is interesting to further study the combined SPECT and CBCT scanner so that it might be used in future generation systems.

## 5 CONCLUSIONS

The x‐ray flat panel detector of a C‐arm CBCT scanner might be used to perform nuclear imaging. The SPECT reconstruction quality will be lower than that achieved by a conventional gamma camera. The flat panel detector approach could, however, be useful in providing a cost‐effective alternative to the purchase of a mobile SPECT scanner for enabling interventional scanning.

## CONFLICT OF INTEREST

This project has received funding from the European Research Council (ERC) under the European Union’s Horizon 2020 research and innovation program grant agreement No. 646734. The proof‐of‐concept experiment was conducted with the assistance of Philips Healthcare (Best, The Netherlands). The authors had full control over the data and information submitted for publication.

## References

[1] Van der Velden S , Kunnen B , Koppert WJC , et al. A dual‐layer detector for simultaneous fluoroscopic and nuclear imaging. Radiology. 2019;290:833–838. 30620257 10.1148/radiol.2018180796

[2] Dietze MMA , Kunnen B , van der Velden S , et al. Performance of a dual‐layer scanner for hybrid SPECT/CBCT. Phys Med Biol. 2019;64:105020. 30947146 10.1088/1361-6560/ab15f6

[3] Dietze MMA , Bastiaannet R , Kunnen B , et al. Respiratory motion compensation in interventional liver SPECT using simultaneous fluoroscopic and nuclear imaging. Med Phys. 2019;46:3496–3507. 31183868 10.1002/mp.13653 PMC6851796

[4] Siewerdsen JH . Cone‐beam CT with a flat‐panel detector: from image science to image‐guided surgery. Nucl Instr Meth Phys Res Sect A. 2011;648:S241–S250. 10.1016/j.nima.2010.11.088 PMC3429946 22942510

[5] Robert C , Montémont G , Rebuffel V , Buvat I , Guérin L , Verger L . Simulation‐based evaluation and optimization of a new CdZnTe gamma‐camera architecture (HiSens). Phys Med Biol. 2010;55:2709–2726. 20400808 10.1088/0031-9155/55/9/019

[6] Robert C , Montémont G , Rebuffel V , Verger L , Buvat I . Optimization of a parallel hole collimator/CdZnTe gamma‐camera architecture for scintimammography. Med Phys. 2011;38:1806–1819. 21626915 10.1118/1.3560423

[7] Suzuki A , Takeuchi W , Ishitsu T , Tsuchiya K , Ueno Y , Kobashi K . A four‐pixel matched collimator for high‐sensitivity SPECT imaging. Phys Med Biol. 2013;58:2199–2217. 23475193 10.1088/0031-9155/58/7/2199

[8] Suzuki A , Takeuchi W , Ishitsu T , et al. High‐sensitivity brain SPECT system using cadmium telluride (CdTe) semiconductor detector and 4‐pixel matched collimator. Phys Med Biol. 2013;58:7715–7731. 24140804 10.1088/0031-9155/58/21/7715

[9] Kasap S , Frey JB , Belev G , et al. Amorphous and polycrystalline photoconductors for direct conversion flat panel x‐ray image sensors. Sensors. 2011;11:5122–5157. 10.3390/s110505112 PMC3231396 22163893

[10] Seco J , Clasie B , Partridge M . Review on the characteristics of radiation detectors for dosimetry and imaging. Phys Med Biol. 2014;59:R303–R347. 25229250 10.1088/0031-9155/59/20/R303

[11] Jan S , Santin G , Strul D , et al. GATE: a simulation toolkit for PET and SPECT. Phys Med Biol. 2004;49:4543–4561. 15552416 10.1088/0031-9155/49/19/007 PMC3267383

[12] Siemens . Symbia S and T System Specifications; 2010.

[13] Cherry SR , Sorenson JA , Phelps ME . Physics in Nuclear Medicine; 2012.

[14] De Jong HWAM , Slijpen ETP , Beekman FJ . Acceleration of Monte Carlo SPECT simulation using convolution‐based forced detection. IEEE Trans Nucl Sci. 2001;48:58–64.

[15] Dietze MMA , Van der Velden S , Lam MGEH , Viergever MA , De Jong HWAM . Fast quantitative reconstruction with focusing collimators for liver SPECT. EJNMMI Phys. 2018;5:28. 30511121 10.1186/s40658-018-0228-5 PMC6277405

[16] Van der Velden S , Dietze MMA , Viergever MA , De Jong HWAM . Fast technetium‐99m liver SPECT for evaluation of the pretreatment procedure for radioembolization dosimetry. Med Phys. 2019;46:345–355. 30347130 10.1002/mp.13253 PMC7379506

[17] Dietze MMA , Kunnen B , Beijst C , Jong HWAM . Adaptive scan duration in SPECT: Evaluation for radioembolization. Med Phys. 2020 10.1002/mp.14095 PMC7317548 32060928

[18] Kunnen B , Dietze MMA , Braat AJAT , Lam MGEH , Viergever MA , De Jong HWAM . Feasibility of imaging ^90^Y microspheres at diagnostic activity levels for hepatic radioembolization treatment planning. Med Phys. 2020;47:1105–1114. 31855282 10.1002/mp.13974 PMC7078991

